# Delayed diagnosis of hemophilia A presenting as chronic joint swelling: a case report

**DOI:** 10.3389/fped.2026.1752234

**Published:** 2026-03-17

**Authors:** Chengxi Yu, Cheng Luo, Lin Yang, Lina Chen

**Affiliations:** 1 Department of Pediatric Pulmonology, West China Second University Hospital, Sichuan University, Chengdu, China; 2 Department of Pharmacy, West China Second University Hospital, Sichuan University, Chengdu, China

**Keywords:** case, coagulation tests, diagnosis, juvenile idiopathic arthritis, pediatric hemophilia

## Abstract

**Background:**

Chronic joint swelling in children usually requires differential diagnosis for juvenile idiopathic arthritis (JIA). Hemophilic arthropathy, despite its rarity, may be an important differential entity, and its identification is challenging in the absence of significant bleeding history.

**Case presentation:**

A 2-year-old boy admitted to our hospital with a two-month history of recurrent left knee swelling. The patient was initially diagnosed with JIA based on the MRI findings of synovitis and joint effusion. No personal or family history of bleeding disorders was noted. The vital diagnostic clue emerged from meticulous history-taking, which revealed prolonged resolution of cutaneous bruising after minor trauma. Coagulation studies showed an isolated prolonged activated partial thromboplastin time of 65.2 s with a positive correction test. Factor VIII activity was severely reduced to 2.8%, which confirmed the diagnosis of moderate hemophilia A. Factor VIII replacement therapy achieved favorable clinical outcomes in this patient.

**Conclusion:**

This case emphasizes that pediatric hemophilia may mimic arthritis and underscores the essential role of thorough history-taking in identifying subtle bleeding tendencies. We recommend including coagulation tests in children with unexplained persistent joint swelling, especially in boys, before initiating treatment for JIA. This strategy helps reduce the risk of misdiagnosis and prevents harmful interventions.

## Introduction

The assessment of chronic joint swelling remains a clinical challenge in pediatrics. Juvenile idiopathic arthritis (JIA), a chronic autoimmune disorder, is among the most common chronic diseases in childhood. It is characterized by synovial inflammation and hyperplasia leading to pathological angiogenesis, pannus formation, progressive articular cartilage destruction, and joint structural damage (1, 2). Despite being a frequent initial diagnosis, JIA still requires careful differential diagnosis. Hematologic disorders including hemophilic arthropathy may present similar clinical features (3). The diagnostic difficulty is particularly in patients with mild or moderate hemophilia where typical bleeding history may be absent. Hemophilic arthropathy is usually polyarticular, but it may present as recurrent monoarthrits affecting the same joint. Ankles are commonly affected in children; knees, elbows and ankles in adolescents and adults (4). Mild hemophilia often remains undiagnosed until uncontrolled postoperative bleeding develops, which may lead to severe complications (5). Maintaining clinical suspicion and establishing early diagnosis are crucial. We report a case of delayed hemophilia diagnosis in a child initially diagnosed as JIA, highlighting the need to include coagulation disorders in the differential diagnosis of pediatric arthropathy.

## Case presentation

**Figure 1:**
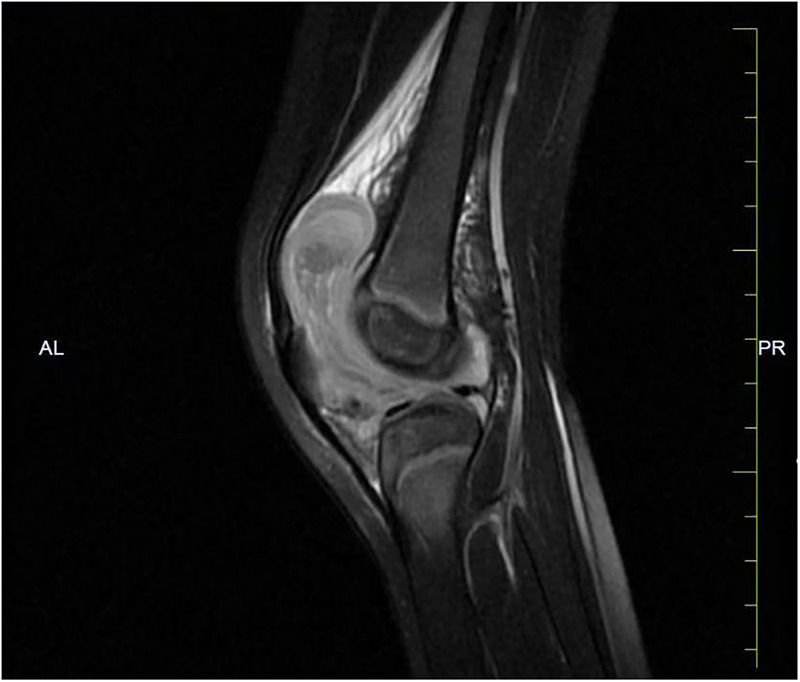
MRI of the left knee showing suprapatellar and joint cavity effusion with synovial hypertrophy and associated soft tissue swelling on T2-FSE imaging.

**Figure 2:**
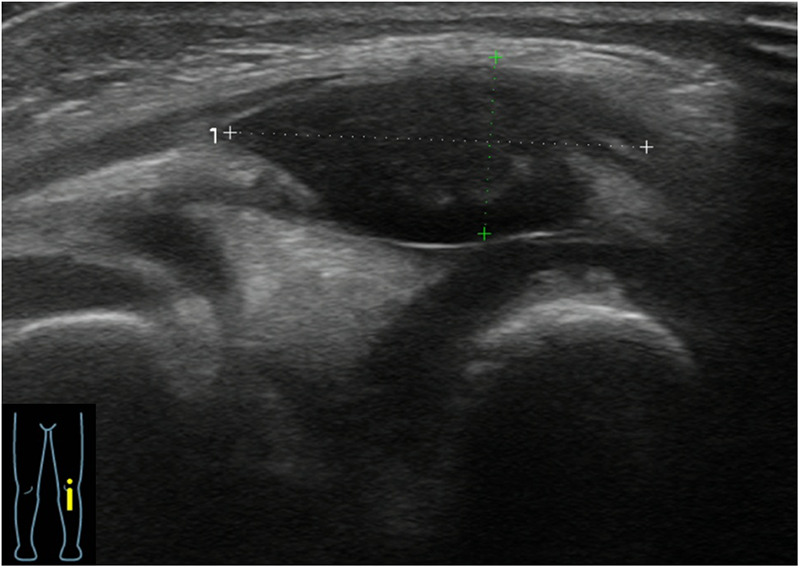
Ultrasonography of the left knee showing a hypoechoic fluid collection with internal and flocculent echoes.

A previously healthy 2-year-old boy was referred to our department for recurrent left knee swelling over two months. The symptoms began two days after prolonged bicycle riding (approximately 50 min), presenting as left knee swelling, pain, and limping. The affected joint exhibited increased local warmth without erythema and no systemic symptoms such as fever and rash. Although the patient's symptoms partially resolved with symptomatic management and restricted activity, the knee swelling persisted. No history of significant trauma, easy bruising, prolonged bleeding, or epistaxis was noted. Family history was unremarkable for bleeding disorders or autoimmune diseases. Persistent joint swelling, combined with MRI findings of suprapatellar and intra-articular effusions as well as left knee soft tissue swelling (Figure 1), led to the suspicion of JIA. Ultrasonography revealed a hypoechoic fluid collection with internal and flocculent echoes in the left knee joint (Figure 2).

On admission, physical examination revealed a well-appearing child with normal vital signs. The left knee exhibited swelling (circumference: 23 cm vs. 19 cm on the right), warmth, and limited range of motion. No redness, swelling, warmth, tenderness, or limited range of motion was observed in other joints. No additional abnormalities detected. Initial laboratory investigations revealed normal blood cell counts, a plasma C-reactive protein (CRP) level of 0.5 mg/L, and an erythrocyte sedimentation rate (ESR) of 17 mm/h. Autoimmune serology (rheumatoid factor, anti-cyclic citrullinated peptide antibodies, antinuclear antibodies) was negative. Tumor necrosis factor-α (TNF-α) was elevated at 12.50 pg/mL (normal range: <8.1 pg/mL), while interleukin (IL)-1β, IL-2R, IL-6, IL-8, and IL-10 were within normal limits. The tuberculosis interferon-gamma release assay (IGRA) was negative, and liver and kidney function tests were unremarkable.

Detailed history-taking revealed no significant bleeding history but the mother reported unusually slow resolution of bruises following minor trauma in the patient. Given the patient's male gender and isolated knee involvement, a bleeding disorder was suspected. Coagulation studies demonstrated a prolonged activated partial thromboplastin time (APTT) of 65.2 s. Knee ultrasound confirmed hemarthrosis. Later, factor assays showed markedly reduced Factor VIII activity (2.8%) with normal levels of Factors IX, XI, II, V, and VII. Immediate APTT correction with normal plasma (29.3 s) indicated a factor deficiency. Further testing identified consistent factor activity across dilutions and undetectable inhibitor levels. Finally, moderate hemophilia A was diagnosed. Factor VIII replacement therapy was effective leading to rapid resolution of knee swelling. The patient was referred to hematology and follow-up revealed normal joint function.

## Discussion

Hemophilia A (factor VIII deficiency) and hemophilia B (factor IX deficiency) are the most common severe congenital coagulation factor deficiencies. Both are X-linked recessive disorders, with males being the predominantly affected population (6). Hemophilia severity is categorized based on residual plasma levels of clotting factor activity (factor VIII or factor IX): mild (5%–40%), moderate (1%–5%), and severe (<1%) (7). Intra-articular bleeding is the most common presentation of hemophilia. Recurrent hemarthrosis initially induces pain and later results in degenerative joint disease, which include synovial hypertrophy, capsular inflammation and retraction, as well as chondral erosion, osteophyte formation, and subchondral cysts (3). Severe hemophilia is easily recognized due to recurrent spontaneous bleeding episodes, while mild hemophilia presents with minimal bleeding that often occurs only after major trauma or surgery and may remain undiagnosed clinically until adulthood (5, 8).

This case highlights the diagnostic challenge of chronic joint swelling. Although JIA is often the initial diagnosis due to its prevalence, hemophilic arthropathy can mimic inflammatory arthritis clinically and radiologically. Moderate hemophilia is characterized by bleeding that occurs primarily in response to recurrent mechanical stress or joint motion, rather than overt spontaneous bleeding. Recurrent hemarthrosis leads to synovial hypertrophy, effusion, and even pseudotumor formation, often presenting on MRI as synovitis indistinguishable from inflammatory arthritis, as reported in previous case studies (3, 8). MRI-detected synovitis stresses the non-specificity of imaging findings. Joint ultrasound may serve as a valuable tool to further differentiate effusion from hemarthrosis (9). Although initial MRI findings mimicked JIA in this patient, careful history-taking of delayed bruise resolution led to prompt diagnosis, thus avoiding unnecessary diagnostic tests and therapeutic interventions. These findings emphasize the critical role of thorough clinical history in the evaluation of complex and atypical cases.

The initiation diagnosis of hemophilia is often delayed, with reported diagnostic delays over 8 years and average age ranging from 5.77 to 34.6 years (10). Central nervous system hemorrhage represents the most severe and functionally disabling manifestation among patients with hemophilia A. Importantly, early identification and timely treatment for hemophilia A are crucial to preventing serious complications (11). The diagnostic delay observed in our case is consistent with several previous reports (4, 5, 12). Several factors contribute to delayed diagnosis in these cases: lack of a bleeding history, clinical overlap with chronic arthritides such as pigmented villonodular synovitis, JIA, or septic arthritis, as well as non-specific inflammatory markers. Notably, these factors are predominantly observed in mild to moderate hemophilia. Hemophilic arthropathy should always be considered in a first episode of joint swelling particularly in male children. Recurrent spontaneous episodes may support an underlying bleeding disorder.

Hemophilia remains frequently misdiagnosed largely due to limited clinical awareness and inadequate testing. In contrast, basic coagulation tests in hemophilia A often show a significantly prolonged APTT, which serves as a useful functional assay (11, 13). Maintaining a high index of clinical suspicion and incorporating coagulation screening (including PT and APTT) into the initial assessment of children with unexplained persistent joint swelling is critical. This low-cost, high-yield approach can identify underlying bleeding disorders and avoid misdiagnosis as chronic inflammatory arthritis.

## Conclusion

This case illustrates that not all joint swelling in children is arthritis. Hemophilic arthropathy can mimic inflammatory arthritis clinically and radiologically, especially when typical bleeding episodes are absent. Careful history-taking and basic coagulation screening are essential in children with unexplained joint swelling, particularly in boys, to ensure timely diagnosis. Improving awareness among clinicians and caregivers is crucial for prompt and appropriate management.

## Data Availability

The original contributions presented in the study are included in the article/Supplementary Material, further inquiries can be directed to the corresponding author.
